# Book Reviews

**Published:** 1892-09

**Authors:** 


THE SCIENCE AND ART OF MIDWIFERY. By WILLIAM THOMPSON LUSK, A. M., M. D., Professor of Obstetrics and Diseases of Women and Children in the Bellevue Hospital Medical College ; Consulting Physician to the Maternity Hospital and to the Foundling Asylum ; Visiting Physician to the Emergency Hospital ; Gynecologist to the Bellevue and to the St. Vincent Hospitals ; Honorary Fellow of the Edinburgh and London Obstetrical Societies ; Corresponding Fellow of the Obstetrical Societies of Paris and Leipzig ; Corresponding Fellow of the Paris Academy of Medicine, etc. New edition. Revised and enlarged, with numerous illustrations. New York : D. Appleton & Co. 1892.

This edition is the fourth of this valuable work. The third edition was issued in 1885, and, up to that date, contained all the advances made in this important department of medicine. The present edition is essentially a new work, having been rewritten to include the still greater advances of the last decade.

Dr. Lusk shows his experience as a teacher in making his work subserve the double purpose of a text-book for the student and a reference book for the practical obstetrician. The arrangement of subjects follow in a natural and logical order. In the first three chapters, the Anatomy of the Internal and External Genitalia, the Development of the Ovum and Fetus are presented. The fourth and fifth chapters treat of the Physiology of Pregnancy, and the following six chapters are devoted to the consideration of Labor. This portion of the volume furnishes a valuable text-book for the student in following the lecturer. The various subjects are treated clearly and concisely. The Physiology and Management of Childbed takes up one chapter, and is full of practical rules and useful suggestions. The author wisely avoids the lax methods, now prevailing, which sanction a free diet after labor, and the too early substitution of the erect for the recumbent position, before involution has been accomplished.

The chapters on the Pathology of Pregnancy are devoted to accidental complications and abnormalities of the uterus, diseases of the decidua and ovum, premature expulsion of the ovum, and extra-uterine pregnancy.

The chapters on Obstetric Surgery include the induction of premature labor, the forceps, and its application, breech presentations, version, craniotomy and embryotomy, and Cesarean section.

The Pathology of Labor takes up eleven chapters, which are devoted to the consideration of the anomalies of the expelling forces, contracted pelves and their treatment, rare forms of pelvic distortion, abnormalities of the sexual organs and of the fetus, eclampsia, post-partum hemorrhage and retained placenta, placenta previa, accidental hemorrhage, inversion of the uterus, rupture of the genital canal, and prolapsus funis.

The last three chapters consider the Diseases of Child-bed, Puerperal Infection and Insanity, Phlegmasia Dolens and Diseases of the Breasts.

The above furnishes an outline of the subjects treated. The volume claims a favorable criticism and warm endorsement. The publishers have shown commendable enterprise in equipping this valuable work with paper and typography that makes it attractive to the reader.

---

First Lines in Midwifery: A Guide to Attendance on Natural Labor for Medical Students and Midwives. By G. Ernest Herman, M. D., F. R. C. P., Obstetric Physician to the London Hospital, and Lecturer on Midwifery; Examiner in Midwifery to the Royal College of Surgeons. In one 12mo volume of 198 pages, with eighty illustrations. Cloth, $1.25. Students' Series of Manuals. Philaphia: Lea Brothers & Co. 1892.

The object of this little book is to give to the student in attendance on his first case of labor such elementary knowledge as is needed for the safe management of natural labor. With a view to adapt the work to midwives, a chapter on anatomy has been prefixed.

The work is an excellent compend for the under-graduate to take in his pocket when attending his first obstetric cases, to refresh his memory while waiting for the delivery, and to give confidence until experience is gained.

**Drinking Water and Ice Supplies and their Relations to Health and Disease.** By T. Michell Prudden, M. D., author of The Story of Bacteria; Dust and Its Dangers, etc. Pp. 148. G. P. Putnam's Sons, New York, 27 W. Twenty-third street; London, 27 King street, Strand. The Knickerbocker Press. 1891.

We are pleased to find in this little volume a fitting complement to Dr. Prudden's former admirable contributions, relative to public health and sanitary measures.

Whether used to allay thirst, in the preparation of food, or as a remedial agent, water has ever been the subject of jealous investigation. By its known or supposed curative qualities in certain classes of diseases, its good name has been made use of by pseudo-scientists and quacks, and while Nature by her wonderful chemistry has provided abundance of salutary sources in aid of medical science, ill-smelling puddles of no value have often been made to do duty in aid of designing avarice.

A great deal of ignorance, too, has been ventilated in the daily press as to the purity or impurity of the water we drink, the solar microscope has given us exhibitions of its fearful fauna, money has been wasted in costly devices for filtering that are more or less useless, warnings of all sorts against water in its natural state have made even men afraid to quench their thirst.

The purpose of the little volume before us is clearly outlined in the first chapter, thus:

> There are a good many fancies and beliefs about drinking-water which are founded on ignorance, perpetuated by indifference, and culminate in serious physical disorders. These the writer wishes to expose and combat. There have been many new and important revelations within the past few years about the relations of good water to good health, and of bad water to varying degrees of bad health. These, with what clearness he can master, the writer purposes to set forth.

After a brief review of the chemical history of water, attention is paid to its geological position, and especially the purifying filtering process which it undergoes in percolating the various strata of the earth, a very plain and easily understood account being given of the way in which this is accomplished. Several chapters are then devoted to the bacteria. Here we are relieved to find that among this large number of minute organisms few are dangerous to health,—indeed, the larger part seem to be beneficial, as they contribute to the destruction of much that would be harmful.

Dr. Prudden says :

It is very unfortunate that the earliest notions which people nowadays get of these minute living things should usually be associated with their relationship to disease.

And again :

There are, indeed, as I have intimated, some very stern realities associated with a few species of bacteria, but they need not overshadow existence, even in crowded towns, if we live up to the light which science is throwing wide abroad in this domain, and do not permit the sanitary affairs of our municipality to fall into the clutches of mercenary political tricksters.

The latter part of the work is devoted to useful directions how to obtain water, the management in regard to wells, their structure and position, etc. The book is written in a pleasant style, wholly free from bewildering technicality, every word with telling effect as well adapted to the simplest as to the most comprehensive understanding.

